# Effects of repetitive transcranial magnetic stimulation on episodic memory in patients with subjective cognitive decline: study protocol for a randomized clinical trial

**DOI:** 10.3389/fpsyg.2023.1298065

**Published:** 2023-11-01

**Authors:** Tianjiao Zhang, Sisi Huang, Qian Lu, Jie Song, Jing Teng, Tong Wang, Ying Shen

**Affiliations:** ^1^Rehabilitation Medicine Center, The First Affiliated Hospital of Nanjing Medical University, Nanjing, China; ^2^Department of Rehabilitation Medicine, The Affiliated Jiangsu Shengze Hospital of Nanjing Medical University, Suzhou, China

**Keywords:** episodic memory, subjective cognitive decline, dorsolateral prefrontal cortex, repetitive transcranial magnetic stimulation, trial protocol

## Abstract

**Introduction:**

Early decline of episodic memory is detectable in subjective cognitive decline (SCD). The left dorsolateral prefrontal cortex (DLPFC) is associated with encoding episodic memories. Repetitive transcranial magnetic stimulation (rTMS) is a novel and viable tool to improve cognitive function in Alzheimer’s disease (AD) and mild cognitive impairment, but the treatment effect in SCD has not been studied. We aim to investigate the efficacy of rTMS on episodic memory in individuals with SCD, and to explore the potential mechanisms of neural plasticity.

**Methods:**

In our randomized, sham-controlled trial, patients (*n* = 60) with SCD will receive 20 sessions (5 consecutive days per week for 4 weeks) of real rTMS (*n* = 30) or sham rTMS (*n* = 30) over the left DLPFC. The primary outcome is the Auditory Verbal Learning Test-Huashan version (AVLT-H). Other neuropsychological examinations and the long-term potentiation (LTP)-like cortical plasticity evaluation serve as the secondary outcomes. These outcomes will be assessed before and at the end of the intervention.

**Discussion:**

If the episodic memory of SCD improve after the intervention, the study will confirm that rTMS is a promising intervention for cognitive function improvement on the early stage of dementia. This study will also provide important clinical evidence for early intervention in AD and emphasizes the significance that impaired LTP-like cortical plasticity may be a potential biomarker of AD prognosis by demonstrating the predictive role of LTP on cognitive improvement in SCD.

**Ethics and dissemination:**

The study was approved by the Human Research Ethics Committee of the hospital (No. 2023-002-01). The results will be published in peer-review publications.

**Clinical trial registration:**

https://www.chictr.org.cn/, identifier ChiCTR2300075517.

## 1. Introduction

Alzheimer’s disease (AD), a neurodegenerative disorder of great concern in the context of the aging population, has come to be viewed not only as an isolated clinical diagnosis but as a multilevel process that changes along a sequential spectrum (Aisen et al., 2017). The earliest clinical manifestation in the spectrum of AD is subjective cognitive decline (SCD), also known as self-experienced memory disturbance without objective cognitive impairment (Wang et al., 2020). SCD is of great value when considered as an elevated risk factor for the development of AD dementia (Wolfsgruber et al., 2017; Wang et al., 2020), given that approximately 14.1% of individuals with SCD develop dementia in 4-year follow-up studies (Mitchell et al., 2014). These pathophysiologic changes occur many years before clinical signs of AD and it is likely that effective therapies at the stage of SCD will have the potential to slow or even halt the progression to AD (Si et al., 2020; Wang et al., 2020). Thus, SCD may be of utmost importance as a time node for early interventions in AD [6]. Based on the accumulating evidence from previous meta-analysis findings, non-pharmacological interventions have been widely used in individuals with SCD (Sheng et al., 2020).

Repetitive transcranial magnetic stimulation (rTMS), as a safe and reliable non-pharmacological intervention, has been shown to result in significant cognitive improvement in AD and mild cognitive impairment (MCI) in many research studies (Lin et al., 2019; Chou et al., 2020; Jiang et al., 2020). To date, rTMS over the left dorsolateral prefrontal cortex (l-DLPFC) has been shown to be an effective method of treatment in AD (Level C of evidence) (Di Lazzaro et al., 2021). The l-DLPFC is the most common choices for single site rTMS stimulation. A number of studies which targeted the l-DLPFC have shown significant improvements in cognitive function scores (Hauer et al., 2019; Lin et al., 2019; Zhang et al., 2022).

Episodic memory (EM) represents the ability to recall and recognize previously encountered objects, people, and events, and serves as a process that is critical for advanced cognitive functions such as judgment and decision-making (Wang, 2021; Lalla et al., 2022). In some studies, EM has been found to be a potentially sensitive indicator for pathological conditions such as AD (Tromp et al., 2015; Xue, 2018; Yu et al., 2021). It is also possible that EM may already be impaired relative to healthy controls (HC) in the SCD stage using the Auditory Verbal Learning Test-delayed recall (AVLT-DR), even when standardized memory tests show no decline (Zhu et al., 2021). Transcranial direct current stimulation (tDCS) and rTMS studies have shown that the dorsolateral prefrontal cortex (DLPFC) region plays an important role in strengthening EM associative memory and recall via reconsolidation in patients with dementia (Solé-Padullés et al., 2006; Vaqué-Alcázar et al., 2021). One previous research has demonstrated that the application of anodal tDCS on the left lateral prefrontal cortex (PFC) enhances pre-existing episode memories, with the effect persisting for a period of 30 days in elderly individuals with SCD (Manenti et al., 2017). Nonetheless, these results demonstrate the ability of the interventions to transiently influence brain function and did not identify them as therapeutic tools for individuals with memory impairment who performed poorly on neuropsychological tests (Solé-Padullés et al., 2006; Vaqué-Alcázar et al., 2021; Cotelli et al., 2022). Thus, to date, the efficacy of rTMS as a therapeutic tool for patients with SCD requires further investigation.

Previous studies have reported the varied neural mechanisms of rTMS for ameliorating cognitive impairment. rTMS may not only regulate the regulation of cortical excitability but may also lead to changes in cerebral blood flow, and neurotransmitters as well as the level of brain derived neurotrophic factor. Most importantly, rTMS may also alter synaptic plasticity and brain networks (Griskova et al., 2006). A Long-term potentiation (LTP) is one of the most extensively studied forms of synaptic plasticity (Wichmann and Kuner, 2022), and is the key cellular basis of the learning and memory process, which can be induced and evaluated simply by transcranial magnetic stimulation (TMS) protocols (Di Lorenzo et al., 2020b; Di Lazzaro et al., 2021). Large studies of patients with AD have demonstrated that changes in the LTP mechanism are linked to memory loss and increased levels of CSF tau. This association is particularly strong when coupled with the apolipoprotein E gene (APOE) ε4 polymorphism and progression of the disease (Francesco and Koch, 2021). Therefore, considering its potential as a biomarker for assessing synaptic impairment, TMS-assessed LTP may provide reliable information about related physio-pathological events in AD. Higher levels of CSF t-Tau of individuals with AD are linked with more powerful inhibition of motor evoked potentials, as induced by the 1 Hz TMS protocol (Koch et al., 2011). Moreover, LTP may be a as a predictive tool for revealing the progression of cognitive decline across the spectrum of AD (Motta et al., 2018). Previous studies have found that participants with MCI and amyloid positivity showed abnormal LTP-like plasticity with poorer memory function (Buss et al., 2020). These findings indicate the potential for utilizing LTP as a prognostic indicator or therapeutic target for the early stages of AD.

It is suggested that impairment to synaptic plasticity plays a crucial role in the development and progression of AD (Walsh and Selkoe, 2004; Shankar et al., 2008). According to Mecca et al. (2020) [^11^C]UCB-J PET technology demonstrated significant synaptic loss caused by AD at neocortical areas such as frontal regions. Another study suggest that cerebellar LTP-like cortical plasticity mechanisms are impaired in AD (Di Lorenzo et al., 2020a). Since the discovery of SCD, neuropathological abnormalities in amyloidosis (e.g., reductions in Aβ42 and increases in amyloid tracer uptake) and neurodegeneration have appeared. The latter includes atrophy in the medial temporal lobes and paralimbic and temporoparietal cortices (Rabin et al., 2017). The loss of long-term EM has not only been referred to as merely local damage to the medial temporal lobes, but also to the malfunction of cortical plasticity on the basis of memory processes (Vidal-Piñeiro et al., 2014). Considering the precocious impairment of plasticity mechanisms in AD, researching synaptic mechanisms in patients who show initial signs of memory deficits can effectively identify early functional anomalies, predict disease progression and evaluate the effectiveness of treatments. We, therefore, decided to study the change in LTP-like plasticity induced by SCD treatment with TMS.

The primary objective of our study is to investigate the efficacy of rTMS on EM in patients with SCD. The Auditory Verbal Learning Test-Huashan version (AVLT-H) will be used as the primary outcome measure. We also explore changes in the both neural and behavioral effects (other cognitive domains) after rTMS interventions. The predictive role of the LTP-like plasticity on cognitive improvement in rTMS-treated SCD patients will be studied. We hypothesized that stimulating the left-DLPFC (l-DLPFC) with 10 Hz rTMS can produce beneficial effects on EM in SCD by enhancing LTP-like plasticity.

## 2. Materials and methods

### 2.1. Study design

**FIGURE 1:**
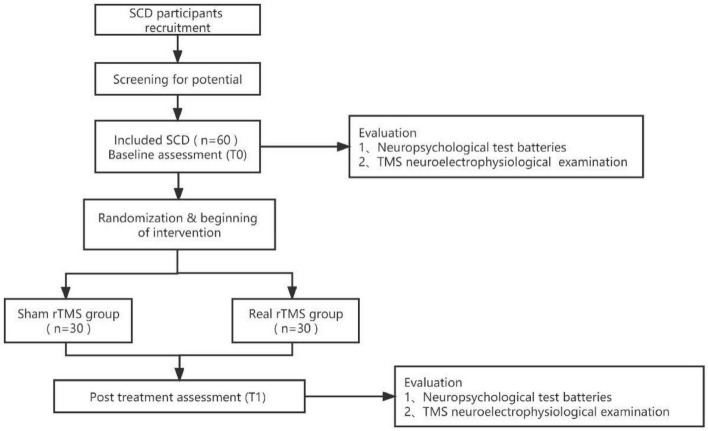
Study flow diagram.

This is a randomized, double-blind, sham-controlled clinical trial, based on the Consolidated Standards of Reporting Trials (CONSORT) statement for non-pharmacologic therapy (Boutron et al., 2008). This study protocol was prepared in accordance with the Standard Protocol Items for Randomized Trials (SPIRIT) statement (Chan et al., 2015). The study was approved by the Human Research Ethics Committee at our hospital (No. 2023-002-01) and was registered with the Chinese Clinical Trial Registry (ChiCTR2300075517). Figure 1 shows details of the study design. Baseline (T0) assessments will assess demographic, behavioral, neurophysiological, and neuroimaging indicators and, following completion of the intervention program (T1), instantaneous outcomes will be measured and compared between the two groups. Patients will undergo a comprehensive clinical investigation including a medical history and a thorough neuropsychiatric assessment exploring cognitive domains (e.g., global cognitive function, language function, executive function, memory, and attention), neuroelectrophysiological examination via TMS before and after completion of the procedure. Cognitive tests and TMS test will be performed in the morning and afternoon on the same day, respectively.

### 2.2. Participants

Enrollment of participants began in September 2023 from memory clinics, or surrounding communities. Right-handed participants will be screened and evaluated by a specialist using comprehensive neuropsychological test batteries at the Department of Rehabilitation Medicine of the Affiliated Jiangsu Shengze Hospital of Nanjing Medical University in China. All participants will sign a written informed consent before the program in accordance with the Declaration of Helsinki.

### 2.3. Eligibility criteria

The inclusion criteria are based on Jessen’s criteria as follows (Jessen et al., 2014; Shen et al., 2022): (1) not meeting the diagnosis of MCI on the standardized neuropsychological tests, including memory, speed/executive function, and language domains (Zhong et al., 2021); (2) self-perceived memory loss for at least 6 months; (3) presence of concern that performance in memory is worse than other people of the same age; (4) 55 to 80 years. The exclusion criteria are as follows: (1) clinical diagnosis of vascular dementia (Modified Hachinski Ischemic Score > 4) or dementia (NINDS-AIREN criteria); (2) drug or alcohol dependence within the last 6 months; (3) presence of severe cardiovascular or cerebrovascular disease or psychiatric disease; (4) contraindications to TMS; (5) treatment with antidepressants, anxiolytics, or central nervous system medications within 3 months prior to the assessments; (6) geriatric depression scale scores ≥ 6.

### 2.4. Sample size

According to Jia et al. (2021), the sample size was calculated using G*Power software, with a Cohen’s effect size of *d* = 0.7979 for rTMS on the 12-word Philadelphia Verbal Learning Test as the primary outcome (Jia et al., 2021). Moreover, alpha and power are set at 0.05 and 80%, respectively. Our study will increase the inclusion number by 10% to compensate for possible patient shedding. The final number of participants will be 30 per group.

### 2.5. Randomization and blinding

The randomization procedure will be performed by a researcher at a 1:1 balanced distribution ratio of (real or sham group) using a web-based randomization tool.^1^ Randomization information will be passed by sealed envelope until completion of the study. One investigator will oversee the random sequence and, at the intervention stage, the trained investigator will set the mode of the stimulus (real or sham group) according to the number on the paper in the envelope. Participants will be identified by codes rather than real names throughout the study. The neuroelectrophysiological assessor and the statistician will be blinded, as they are independently involved in the assessment or data analysis process, respectively.

### 2.6. Intervention

Repetitive transcranial magnetic stimulation (rTMS) treatment will be delivered using a D-MT500 magnetic biphasic stimulator (Neurosoft Ltd., Russia; peak magnetic field = 4T) equipped with an eight-shaped coil (AFEC-02-100-C; Neurosoft Ltd., Russia; diameter = 100 mm). Participants will randomly receive either rTMS or sham treatments over the l-DLPFC. The stimulation site is situated at the F3 point using the Bean F3 method as per the international 10–20 system for standardized placement of electroencephalogram (EEG) electrodes (Beam et al., 2009). Each treatment stimulation session consists of 10 Hz rTMS with 5 s train duration (50 pulses per train), and intertrain intervals of 10 s. In total, there are 40 trains with 2,000 pulses per day, 10 min each time, for 5 consecutive days per week for 4 weeks. Intensity will be set at 90% of the resting motion threshold (RMT) for each participant, defined as the minimum single-pulse intensity that triggers motor evoked potential (MEP) (not less than 50 μV) in at least 5 of 10 hotspots in the contralateral abductor pollicis brevis (APB) trial. The sham procedure is provided by the same device through a self-contained sham stimulus procedure. And the sessions are matched in all subjects.

The electromyography (EMG) system (Neuro-MEP-Micro, Russia) will be used to record the MEPs of the right-hand APB through surface Ag-AgCl electrodes. Through visual and EMG monitoring, entire relaxation of the muscle will be ensured. The coil is first placed on the left M1, and the handle placed backward at 45° against the midline of the sagittal plane of the brain. To determine the hotspot, where the lowest intensity induces the highest MEP amplitude, the coil is moved every 0.5 cm each time around the presumed scope. If the hotspot cannot be confirmed within 10 stimuli, the coil is shifted to the next location. Once the motor hotspot has been identified, the RMT will be determined.

### 2.7. Outcome measurements

#### 2.7.1. Primary outcomes

The Auditory Verbal Learning Test-Huashan version (AVLT-H) assessment will be used as the primary outcome to assess EM for our study. The AVLT-H assesses several aspects of verbal EM through a list of 12 words, such as short or long-term delayed recall and recognition. It has been widely used as a semantic categorization memory test in mainland Chinese populations (Li et al., 2016). The AVLT-H scores include AVLT-H immediate recall total score (AVLT-H-IR-S), AVLT-H short-term delayed recall score (AVLT-H-SR-S) with a 5-min delay time, AVLT-H long-term delayed recall score (AVLT-H-LR-S) with a 20-min delay time, AVLT-H total, and AVLT-H recognition score (AVLT-H-REC-S) (Zhao et al., 2012). This test has been proved to be a sensitive diagnostic evaluation of cognitive impairment (Li et al., 2016).

#### 2.7.2. Secondary outcomes

Other cognitive domain examinations and LTP-like cortical plasticity will also be measured. Overall cognitive function will be assessed using scores from the Montreal Cognitive Assessment Test (MOCA) as well as the Mini-Mental State Examination (MMSE). In addition, the Wechsler Memory Scale -Logical Memory Test (WMS-LM) and digit span test (DST) will be used to assess memory function and attention, respectively. Language function is measured using the scores of the Animal Fluency test (AFT) and Boston Naming Test China version (BNT-C) test. Measures of executive function include the symbol digitized modality test (SDMT) and the trail making test (TMT) parts A and B (Zhong et al., 2021). Time to completion of TMT A and TMT B will be logged and analyzed. In addition, all participants will be asked to complete two computer experiments conducted using E-Prime 2.0 software (Psychology Software Tools Inc., Pittsburgh, PA, United States). The n-back (*n* = 1) task is used to evaluate working memory, while the Go/No-Go task is used to assess inhibitory control ability (Borgwardt et al., 2008; Kumar et al., 2017). The accuracy and response time will be recorded.

The changes of the MEP amplitude will be measured to assess LTP-like cortical plasticity. Twenty consecutive MEPs of 5-s intervals will be evoked by single-pulse TMS at the left motor hotspot of 120% RMT intensity, and the peak-to-peak average value recorded. The whole LTP-like plasticity assessment includes five time points, whereby two baselines before and 5, 10, and 30 min after the intermittent theta burst stimulation (iTBS) protocol. Two baseline measurements are performed 10 min apart, and the subsequent iTBS paradigm is applied if the variation between the two average measurements is < 15%. The iTBS protocol consists of a burst of three stimuli at 50 Hz and repeated at 5 Hz. A 2-s train of this protocol will be repeated every 10 s for a total of 192 s (600 pulses) with 80% RMT over the left hotspot (Huang et al., 2005; Yu et al., 2020).

### 2.8. Data analysis

SPSS V.23.0 will be used to analyze the data, and levels of statistical significance will be set at *p* < 0.05. For descriptive statistics, the Shapiro–Wilk test will be applied to check for normal probability prior to data entry. Data from the normal distribution will be reported as mean and standard deviation, whereas medians with interquartile ranges will be used to express the non-normal distribution. Categorical variables will be described as frequency as a function of percent. Demographic characteristics and baseline variables will be compared between the two groups using independent samples *t*-test or non-parametric Mann–Whitney test for continuous data, and comparisons of categorical variables using chi square or Fisher’s exact tests. Based on the existing literature, we can determine whether the unbalanced baseline data affect the results and, if so, conduct covariance analysis using the baseline data as covariates to control for the effect of potential confounders. The experiment will be analyzed using intention-to-treat (ITT) and missing data will be interpolated using multiple imputation method.

For normally distributed data, a repeated-measures analysis of variance (ANOVA) will be conducted to evaluate changes in treatment effect between and within groups (group: real vs. sham rTMS) (time: pre-vs. post-treatment). Alternatively, for non-normally distributed variables, we will perform the Wilcoxon signed rank test for within group comparisons, and the Mann–Whitney test will be applied to compare the effect between groups at each time point. In addition, we also aim to use a repeated-measures ANOVA to examine LTP-like plasticity as a function of percent change at four time points (last baseline, 5, 10, and 30 min after iTBS) between the two groups. *Post hoc* comparisons between groups will be used using the Bonferroni correction method. The generalized linear model (GLM) will be considered if the data is not normally distributed. To investigate the relationship between brain measurements and clinical cognitive function characteristics, we will perform correlation analyses by repeated measures linear regressions.

### 2.9. Safety

Adverse events are any negative experiences, such as headache, vertigo, seizure, etc., that occur in a patient undergoing TMS. Any adverse event that happens during or immediately after stimulation by TMS should be reported. Participants will be screened strictly according to the inclusion and exclusion criteria to minimize the risk of adverse events. All adverse events will be recorded by the study staff on a case report form, primarily including the date, duration and severity. If a serious event occurs, it will be reported immediately to the principal investigator and the ethics committee. All participants were requested to complete TMSens_Q, a questionnaire designed to report unintended effects of rTMS at the end of every session (Giustiniani et al., 2022).

## 3. Discussion

We aim to assess the cognitive effect of 10 Hz rTMS stimulation specifically over the l-DLPFC. Markedly, non-drug interventions are more acceptable than pharmaceutical treatment for patients with milder symptoms of SCD. Current meta-analyses on traditional interventions (e.g., physical activity, education programs, and cognitive training) for SCD show that cognitive interventions have a modest effect in improving objective cognitive performance; however, in specific cognitive domains, the small improvements are still doubtful (Smart et al., 2017; Bhome et al., 2018). Therefore, we suggest that exploring a novel and promising treatment method such as TMS is of great value for the cognitive enhancement of SCD patients.

The DLPFC plays an essential role in governing EM binding and encoding robust representations (Wang et al., 2018). It is also the most prevalent and effective stimulation target to enhance cognitive function in MCI and AD (Zhang et al., 2022). The stimulation over this area may facilitate the top-down activation of semantic knowledge (Higo et al., 2011). This view agrees with some tDCS studies that show that the left superior parietal and the dorsolateral and anterior PFC regions are more intensely involved in the retrieval process of EM memory, and that stimulation over them facilitates verbal memory retrieval performance (Manenti et al., 2013; Vaqué-Alcázar et al., 2021). Turriziani et al. (2019) already found that the 1-Hz rTMS of the right DLPFC could improve EM performance compared to the sham rTMS and left DLPFC rTMS in AD patients. Accordingly, we decided to investigate whether the excitatory rTMS stimulation over the left DLPFC has the same promotion effect in our protocol. One study reported that higher activation was observed in the DLPFC of MCI subjects. The overactivity may represent a compensatory mechanism that allows these patients to perform better (Gigi et al., 2010). rTMS has the potential to recruit compensatory networks, such as the right prefrontal regions, which participate in memory coding processes (Solé-Padullés et al., 2006). High-frequency rTMS over the l-DLPFC could induce electrophysiological excitatory effects, and increase the efficiency of resource deployment in the prefrontal cortex (Li et al., 2017). For instance, 5 Hz rTMS has been proven to increase the locally successful correlated activity, like the local strength of PFC connectivity (Davis et al., 2017). Furthermore, some studies have found that the additional recruitment of neural resources in the DLPFC region was considered to compensate for the reduction in hippocampal activation in SCD patient or hippocampus atrophy correlated with AD (Erk et al., 2011).

The cortical plasticity is thought to be an important mechanism for information processing in brain neural networks during motor and skill learning (Haley and Maffei, 2018; Mansvelder et al., 2019). LTP could also account for the neurophysiological basis of highly connected node formation. Based on the outcomes of our unpublished research, we have found that LTP-like cortical plasticity was significantly reduced in an SCD group when compared with an HC group. High-frequency rTMS can excite neurons directly, and correspondingly lower the threshold for synaptic transmission, making the synapse quite active and increasing synaptic connections. Potentially, high-frequency pulses of rTMS could generally induce LTP and restore cortical plasticity (Suppa et al., 2015). Li et al. (2021) reported that the cognitive improvement in patients with AD which correlated with changes in LTP was significant after 6 weeks of treatment with 20 Hz rTMS. However, few studies have yielded the effects of rTMS on LTP-like plasticity in SCD. Therefore, the present study will measure motor cortex plasticity as a proxy for the general form of cortical plasticity to examine the effect.

In this protocol, we assess other domains of cognitive function except for the EM. The small degree of progress in these domains also shows important clinical implications. The results of this trial may provide a significant improvement in cognitive deficits among SCD. Because the effective intervention at the SCD stage could increase the potential for disease reversal, it is particularly important to explore a feasible method thereof. If the results are positive, as expected, this study will shed light on a new direction in cognition management in SCD.

## Ethics statement

The study was approved by the Human Research Ethics Committee of the Affiliated Jiangsu Shengze Hospital of Nanjing Medical University (No. 2023-002-01). The studies were conducted in accordance with the local legislation and institutional requirements. The participants provided their written informed consent to participate in this study.

## Author contributions

TZ: Visualization, Writing–original draft. SH: Project administration, Writing–original draft. QL: Supervision, Writing–review and editing. JS: Writing–original draft. JT: Writing–original draft. TW: Conceptualization, Writing–review and editing. YS: Conceptualization, Funding acquisition, Methodology, Writing–review and editing.

## Footnotes

1
http://www.randomization.com
